# Consistency in electronic portal imaging registration in prostate cancer radiation treatment verification

**DOI:** 10.1186/1748-717X-1-37

**Published:** 2006-09-19

**Authors:** Eric Berthelet, Pauline T Truong, Sergei Zavgorodni, Veronika Moravan, Mitchell C Liu, Jim Runkel, Bill Bendorffe, Dorothy Sayers

**Affiliations:** 1 Radiation Therapy Program, Vancouver Island Centre, British Columbia Cancer Agency, Victoria, BC, Canada; 2 Radiation Therapy Program, Fraser Valley Centre, British Columbia Cancer Agency, Surrey, BC, Canada; 3 University of British Columbia, Victoria, BC, Canada; 4 Population and Preventive Oncology, British Columbia Cancer Agency, Vancouver, BC, Canada

## Abstract

**Background:**

A protocol of electronic portal imaging (EPI) registration for the verification of radiation treatment fields has been implemented at our institution. A template is generated using the reference images, which is then registered with the EPI for treatment verification. This study examines interobserver consistency among trained radiation therapists in the registration and verification of external beam radiotherapy (EBRT) for patients with prostate cancer.

**Materials and methods:**

20 consecutive patients with prostate cancer undergoing EBRT were analyzed. The EPIs from the initial 10 fractions were registered independently by 6 trained radiation therapist observers. For each fraction, an anterior-posterior (AP or PA) and left lateral (Lat) EPIs were generated and registered with the reference images. Two measures of displacement for the AP EPI in the superior-inferior (SI) and right left (RL) directions and two measures of displacement for the Lat EPI in the AP and SI directions were prospectively recorded. A total of 2400 images and 4800 measures were analyzed. Means and standard deviations, as well as systematic and random errors were calculated for each observer. Differences between observers were compared using the chi-square test. Variance components analysis was used to evaluate how much variance is attributed to the observers. Time trends were estimated using repeated measures analysis.

**Results:**

Inter-observer variation expressed as the standard deviation of the six observers' measurements within each image were 0.7, 1.0, 1.7 and 1.4 mm for APLR, APSI, LatAP and LatSI respectively. Variance components analysis showed that the variation attributed to the observers was small compared to variation due to the images. On repeated measure analysis, time trends were apparent only for the APLR and LatSI measurements. Their magnitude however was small.

**Conclusion:**

No clinically important systematic observer effect or time trends were identified in the registration of EPI by the radiation therapist observers in this study. These findings are useful in the documentation of consistency and reliability in the quality assurance of treatment verification of EBRT for prostate cancer.

## Background

In the planning and delivery of external beam radiotherapy (EBRT) for prostate cancer, several potential sources of variability have been documented. Prostate organ motion between [1-17] and during fractions [6-8] has been described by several authors. Variations in organ contouring using different imaging modalities [10-12] and variations in interobserver measures have also been reported [18,19].

Electronic portal imaging (EPI) enables the verification of treatment fields during a course of EBRT. Several protocols of EPI are currently in use in various institutions and vary mainly in terms of the number, timing, and frequency of images acquired over a treatment course. Other potential sources of variability in the treatment verification process include the type of correction with on-line or off-line protocols which represent in fact two different strategies to reduce variability [5-7]. While EPI protocols may be diverse among institutions, the common goal of treatment delivery is to ensure accuracy and consistency throughout the process of image verification.

Few reports from the literature have specifically addressed interobserver variability in portal images verification. In a study of 16 observers of different professional disciplines (radiation oncologists, radiation therapists and physicists) using images of various anatomical sites and a 5-point scale to assess conformity between simulator films and portal images, Bissett et al. demonstrated significant inconsistencies between observers [20]. In another series of electronic portal imaging verification in 18 prostate cancer patients, Dalen et al. reported intraclass correlation coefficients (ICC) consistent with significant agreement between radiation oncologists (ICC 0.58) and radiation therapists (ICC 0.72) [2]. Lewis et al. reported good agreement in a study of 9 observers matching a total of 17 images of pelvic radiotherapy portals [9]. However, there are few data from other institutions to corroborate these findings. Within the framework of an effective EPI protocol, the present report focuses on interobserver consistency associated with EPI registration performed by 6 trained radiation therapists on a cohort of 20 prostate cancer patients undergoing daily EPIs during the first ten fractions.

## Methods

### EPI protocol

The protocol for the verification of treatment fields during EBRT for prostate cancer employed at this institution consisted of amorphous silicon EPI acquisition daily during the first 3 days of treatment. These images are then registered to a template generated from the reference images or digitally reconstructed radiographs (DRR). The registration process is based on anatomy matching of the EPI to the reference DRR. On the AP images the key structures are the superior and inferior pubic rami, the pubic symphysis and the obturator foramen. On the Lat images the key structures are the pubic symphysis, the femoral head and the acetabulum. For each fraction, reference images and EPIs are generated for the Anterior-Posterior (AP) and Left Lateral (Lat) beam incidences. The registration process yields 2 measures of displacement of the isocentre for each beam incidence: Superior-Inferior (SI) and Left-Right (LR) directions for the AP images; and SI and Anterior-Posterior (AP) directions for the Lat images. Based on a review of the literature, tolerance of displacements was set at 5 mm. Any single value of displacement greater than twice the tolerance limit (2 × 5 mm = 10 mm), will lead to an off-line correction and a repeat EPI. The values of displacement calculated for the first three fractions are averaged. If this 3-day average exceeds the set tolerance of 5 mm, an off line correction is also applied and the EPI is repeated. For the AP images, directions of displacements are as follows: superior/inferior = +/-; right/left = +/-. For the Lat images: superior/inferior = +/-; anterior/posterior = +/-. To examine possible time trends, EPIs were obtained daily during the first 10 fractions of each course of EBRT. Ten fractions were examined since previous series have suggested that the position of early fractions may not be representative of the overall systematic error as later fractions [21].

### Patients

Twenty consecutive patients with prostate cancer undergoing radical EBRT over at least 6 weeks were analysed for this study. The AP and Lat EPI of the first ten fractions were registered by 6 trained radiation therapists. Measures of displacements in the SI and LR directions for the AP images and SI and AP directions for the Lat images were independently recorded by the 6 observers, all blinded to each other's results. This yielded a total of 2400 registrations and 4800 values of displacement for the analysis.

### Statistics

Mean displacement values and their corresponding standard deviation were calculated for the whole group and the 6 observers individually. Inter-observer variation was assessed by calculating the standard deviation of the six observers' measurements within each image. The sources of variation in measurements of displacement between the observers and the images were compared using variance components analysis. Time trends were estimated using repeated measures analysis. Random and systemic deviations were subsequently calculated for each observer in accordance to previously published definitions [7]

Random error was defined as variations between fractions during a treatment series, was determined by calculating the spread (1 SD) of differences around the corresponding mean in each patient and then calculating the average of these SDs for the whole group.

Systematic error was defined as deviations between the planned position and the average patient position over the treatment course, were obtained by calculating the mean displacement per patient and then the SD of all patients' means.

## Results

Descriptive statistics of the measurements are presented in Table 1. Errors of a larger magnitude were identified at the beginning of treatment in a few patients which yielded values of maximum or minimum displacements close to or > +/-10 mm.

**Table 1:** Mean, standard deviations and range of displacement measures for the entire study cohort

**Image and Direction of Displacement**	**Number of Displacement Measures**	**Mean Displacement(mm)**	**Standard Deviation (mm)**	**Range of Displacement**
**AP LR**	1200	-0.99	2.66	-9.70 to 5.30
**AP SI**	1200	-1.06	2.44	-11.60 to 18.30
**Lat AP**	1200	0.79	3.16	-7.60 to 13.60
**Lat SI**	1200	-0.52	2.71	-10.60 to 16.40

Abbreviations: AP LR = anterior-posterior image, left-right direction; AP SI = anterior-posterior image, superior-inferior direction; Lat AP = lateral image, anterior-posterior direction; Lat SI = lateral image, superior-inferior direction

An alternate approach to evaluate consistency in measurements is to calculate the individual mean values of the 6 observers and their corresponding standard deviation (SD) for each of the 200 images and the four directions of displacement. These results are presented in Table 2. For the entire group of 6 observers, the individual means range from -1.61 mm to 1.04 mm, while the SD range from 2.21 to 3.59 mm respectively.

**Table 2:** Mean displacement measures by each observer for all patients and fractions

		**Observer**					
		**1**	**2**	**3**	**4**	**5**	**6**

**Image and Direction of Displacement**	**Number of measures**	**Mean in mm (SD)**	**Mean in mm (SD)**	**Mean in mm (SD)**	**Mean in mm (SD)**	**Mean in mm (SD)**	**Mean in mm (SD)**

**AP LR**	200	-0.88 (2.67)	-0.97 (2.69)	-1.14 (2.74)	-1.17 (2.59)	-1.01 (2.72)	-0.80 (2.52)
**AP SI**	200	-1.44 (2.53)	-0.58 (2.31)	-0.95 (2.44)	-0.76 (2.48)	-1.04 (2.21)	-1.61 (2.51)
**Lat AP**	200	0.67 (3.32)	0.37 (2.87)	1.04 (3.59)	0.78 (2.67)	1.03 (3.43)	0.87 (2.99)
**Lat SI**	200	-0.83 (3.17)	-0.63 (2.49)	-0.45 (2.79)	-0.20 (2.31)	-0.45 (2.72)	-0.55 (2.69)

In order to assess the impact of displacement on previously set tolerance limits, we calculated the proportion of measurements within 3 mm and 5 mm from zero for each observer. Overall proportions are presented in Table 3. This assumes that the ideal displacement measurement is equal to zero. The values of 3 mm and 5 mm were selected according to the EPI guidelines currently in effect at our institution. This calculation provides an estimate of the proportion of fractions that would require a correction based on a daily online EPI protocol. Reducing the level of tolerance from 5 mm to 3 mm increases the number of corrections that need to be applied. Differences between observers in meeting tolerance limits were examined using the chi-square test. The proportion of measurements within +/- 3 mm and +/- 5 mm from zero varied significantly between observers for all measures except for the measures of AP images in the LR direction. While the reason for this is unclear, we note that proportion of agreement between observers for these measures had the smallest range among the 6 observers (range 69.5–79.5% = 10% for APLR-3 mm and range 91–93% = 2% for APLR-5 mm, respectively). This suggests that there is less variation between observers for the APLR measurement for the APLR measurement for the conditions stated above. This may also indicate that a discrepancy in this direction and incidence is more readily visualized and agreed upon by a group of observers.

**Table 3:** Proportions of measurements within +/-3 mm and +/-5 mm from 0 and chi-square analysis of variations between observers

**Image and Direction of Displacement**	**% Measurements within +/- 3 mm**	**P value**	**% Measurements within +/- 5 mm**	**P value**
**AP LR**	73.8	.26	92.0	.97
**AP SI**	81.3	.0004	95.3	.0049
**Lat AP**	66.8	.002	87.6	.0003
**Lat SI**	78.2	< .0001	94.0	.0005

To further assess the level of agreement between observers we calculated the standard deviation of the six observers' measured displacement for each image. The results are presented in table 4. The lowest inter-observer variation was for the AP images having a mean SD of 0.7 and 1.0 for the LR and SI directions respectively. Lateral images showed a wider level of interobserver variation with mean SD of 1.7 and 1.4 for the AP and SI directions respectively

**Table 4:** Estimated variance attributed to observers and images using variance components analysis

**Image and Direction of Displacement**	**No of images**	**Mean within-image std dev (mm)**	**Range of within-image std dev (mm)**
**AP LR**	200	0.7	0.1 to 3.5
**AP SI**	200	1.0	0.2 to 2.5
**Lat AP**	200	1.7	0.6 to 4.5
**Lat SI**	200	1.4	0.3 to 4.0

The contribution of the observers and the images to the overall variability in measured displacement was estimated using an analysis of variance component. The results are presented in table 5. The variance between images is considerably larger than that of the observers for all directions of displacement. This indicates that the interobserver variability is very small and contributes little to the overall variability in the measured displacements.

**Table 5:** Sources of variation in measurements of displacement expressed as the variance.

	**Variance**	
**Image and Direction of Displacement**	**Observers**	**Images**

**AP LR**	0.02	6.34
**AP SI**	0.15	4.89
**Lat AP**	0.05	6.58
**Lat SI**	0.03	4.92

The presence of time trends was examined using repeated measure analysis. Time was used as a continuous variable in the model and t-test was used to assess statistical significance. The results are presented in Table 6. Time trends were observed for the APLR and Lat SI measurements (p < 0.001 and p = 0.003 respectively). The magnitude of theses trends were however small (0.09 mm/fraction for APLR and -0.067 mm/fraction for Lat SI).

**Table 6:** Estimated time trends and their significance from repeated measures analysis

**Image and Direction of Displacement**	**Estimated Time Trend and 95% CI**	**P-value for Time Trend**
**AP LR**	0.090 (0.049, 0.130)	< 0.001
**AP SI**	-0.029 (-0.068, 0.009)	0.139
**Lat AP**	-0.017 (-0.067, 0.033)	0.511
**Lat SI**	-0.067 (-0.111, -0.023)	0.003

Table 7 presents the systematic and random errors in displacements calculated for each observer for the four possible displacements. Systematic errors were the largest in the Lat AP measurements, while random errors were the largest in the AP SI measurements.

**Table 7:** Systematic and random errors of each observer for each measurement

**(a) Systematic errors in mm**				
**Observer**	**AP LR (mm)**	**AP SI (mm)**	**Lat AP (mm)**	**Lat SI (mm)**

1	1.8	1.6	2.6	2.1
2	1.8	1.3	2.4	1.5
3	1.8	1.4	3.1	1.8
4	1.7	1.4	2.3	1.5
5	1.8	1.3	2.9	1.6
6	1.5	1.3	2.2	1.7

**(b) Random errors in mm**				

**Observer**	**AP LR**	**AP SI**	**Lat AP**	**Lat SI**

1	2.0	1.7	2.1	2.2
2	2.0	1.6	1.6	1.6
3	2.1	1.7	1.9	2.0
4	1.9	1.7	1.5	1.5
5	2.0	1.5	2.0	1.9
6	2.0	2.0	2.0	1.9

## Discussion

Several protocols of EPI verification have been described in the literature [5-7].

The current study specifically assesses interobserver consistency in the EPI registration within an institutionally-defined EPI protocol.

In this protocol, the EPI registration is performed by trained radiation therapists who are responsible to assess treatment accuracy. The analysis was intended to verify that consistency can be achieved among the individuals independently performing the measures.

There are few studies available from the literature dealing with interobserver variation in EPI registration. In a study by Dalen et al. investigating the concordance of approval between groups of radiation oncologists and radiation therapists, no statistically significant differences between the two groups was demonstrated [2]. In this study, results were analyzed using intraclass correlation coefficients (ICC). This method is often use when several observers are measuring a common parameter. A high ICC however, does not imply agreement on all measurements. Hence, this method of comparing observers should be weighed against the goal of the comparison itself, or, in this instance, the accepted variation or tolerance between measurements. For example, if observer 1 measures a displacement of 1 mm on 10 consecutive fractions while observer 2 measures a displacement of 4 mm on the same 10 consecutive fractions, a correlation coefficient of 1 will be obtained. Yet, the measurements are different and if a tolerance is set at 3 mm, measurements by observer 1 would be considered within acceptable limits while those of observer 2 would require corrections for exceeding the tolerance limits.

Lewis et al. [9] investigated variability among 9 observers in assessing patient movement during pelvic EBRT. These authors demonstrated that interobservers variability may be as low as < 1 mm. Similarly, our analysis showed that an observer effect was present for only 1 of the 4 measurements and a mean difference of only 1 mm was noted between the 2 observers that differed the most in their measurements.

Our center has recently moved toward a fiducial marker protocol for EPI registration in patients receiving EBRT for prostate cancer. The use of gold fiducial markers has been shown to be a feasible and effective method of tracking prostate motion during treatment [3-17]. Nederveen et al. showed that the application of a marker-based verification system can reduce systematic errors when compared to the use of bony anatomy alone [11]. In another study by Ullman et al., high intra- and inter-radiation therapist reproducibility was demonstrated in daily verification and correction of isocenter positions relative to fiducial markers. In this study, using a 5 mm threshold, only 0.5% of treatments required shifts due to intra- or inter-observer error [17]. Analysis of our institution's data using fiducial marker is ongoing to potentially corroborate these results. Although the use of fiducial markers implanted in the prostate is increasingly adopted as a standard in some centres, there remains a large proportion of centres worldwide that continues to rely on bony landmarks in image verification for prostate cancer treatment. In this context, we believe that information regarding the interobserver variability of EPI verification using bony anatomy still provides an important measure of quality assurance. Furthermore, the accuracy of bony anatomy matching remains an important factor since pelvic treatment continues to rely on bony anatomy rather than prostate position [12].

## Conclusion

This study demonstrated significant consistency among trained radiation therapists in EPI registration for treatment verification of radical prostate cancer EBRT. No significant systematic observer effect and no systematic time trends were identified. These findings may serve as measures of quality assurance of the institutional verification protocol.

## Competing interests

The author(s) declare that they have no competing interests.

## Authors' contributions

EB conceived and designed the study, supervised data acquisition and data analysis, drafted the manuscript. PTT participated in study design, data interpretation, manuscript drafting and revising for intellectual content. SZ participated in study design, data interpretation and manuscript revising for intellectual content. VM performed data analysis, data interpretation, and manuscript review and revision. JR, BB, and DS performed data acquisition, data interpretation, manuscript review and revision for intellectual content. All authors have given final approval of this submitted version.

## References

[B1] Crook JM, Raymond Y, Salhani D, Yang H, Esche B (1995). Prostate motion during standard radiotherapy as assessed by fiducial markers. Radiother Oncol.

[B2] Dalen I, Isaksen KE, Storetvedt LH, Berget E, Engeseth GM, Helle SI, Johannessen DC (2002). Do oncologists and radiation technologists evaluate clinical portal images equally? An interobserver reliability study. Proceedings of the 7th International Workshop on Electronic Portal Imaging EPI2K2 Vancouver BC.

[B3] Dehnad H, Nederveen AJ, van der Heide UA, van Moorselaar RJA, Hofman P, Lagendijk JJW (2003). Clinical feasibility study for the use of implanted gold seeds in the prostate as reliable positioning markers during megavoltage irradiation. Radiother Oncol.

[B4] Fiorino C, Reni M, Bolognesi A, Cattaneo GM, Calandrino R (1998). Intra-and inter-observer variability in contouring prostate and seminal vesicles: implications for conformal treatment planning. Radiother Oncol.

[B5] Herman MG, Kruse JJ, Hagness CR (2000). Guide to clinical use of electronic portal imaging. J Appl Clin Med Phys.

[B6] Huang E, Dong L, Chandra A, Kuban DA, Rosen II, Evans A, Pollack A (2002). Intrafraction prostate motion during IMRT for prostate cancer. Int J Radiat Oncol Biol Phys.

[B7] Hurkmans CW, Remeijer P, Lebesque JV, Mijnheer BJ (2001). Set-up verification using portal imaging; review of current clinical practice. Radiother Oncol.

[B8] Kitamura K, Shirato H, Seppenwoolde Y, Onimaru R, Oda M, Fujita K, Shimizu S, Shinohara N, Harabayashi T, Miyasaka K (2002). Three-dimensional intrafractional movement of prostate measured during real-time tumor-tracking radiotherapy in supine and prone treatment positions. Int J Radiat Oncol Biol Phys.

[B9] Lewis DG, Ryan KR, Smith CW (2005). Observer variability when evaluating patient movement from electronic portal images of pelvic radiotherapy fields. Radiother Oncol.

[B10] Narayana V, Roberson PL, Pu AT, Sandler H, Winfield RH, McLaughlin PW (1997). Impact of differences in ultrasound and computed tomography volumes on treatment planning of permanent prostate implants. Int J Radiat Oncol Biol Phys.

[B11] Nederveen AJ, Dehnad H, van der Heide UA, van Moorselaar RJA, Hofman P, Lagendijk JJW (2003). Comparison of megavoltage position verification for prostate irradiation based on bony anatomy and implanted fiducials. Radiother Oncol.

[B12] Parker CC, Damyanovich A, Haycocks T, Haider M, Bayley A, Catton CN (2003). Magnetic resonance imaging in the radiation treatment planning of localized prostate cancer using intra-prostatic fiducial markers for computed tomography co-registration. Radiother Oncol.

[B13] Rasch C, Barillot I, Remeijer P, Touw A, van Herk M, Lebesque JV (1999). Definition of the prostate in CT and MRI: a multiobserver study. Int J Radiat Oncol Biol Phys.

[B14] Roach M, Desilvio M, Lawton C, Uhl V, Machtay M, Seider MJ, Rotman M, Jones C, Asbell SO, Valicenti RK, Han S, Thomas CR, Shipley W (2003). Phase III Trial comparing whole pelvis versus prostate-only radiotherapy and neoadjuvant versus adjuvant combined androgen suppression: Radiation Therapy Oncology Group 9413. J Clin Oncol.

[B15] Shirato H, Harada T, Harabayashi T, Hida K, Endo H, Kitamura K, Onimaru R, Yamazaki K, Kurauchi N, Shimizu T, Shinohara N, Matsushita M, Dosaka-Akita H, Miyasaka K (2003). Feasibility of insertion/implantation of 2.0-mm-diameter gold internal fiducial markers for precise setup and real-time tumor tracking in radiotherapy. Int J Radiat Oncol Biol Phys.

[B16] Wu J, Haycocks T, Alasti H, Ottewell G, Middlemiss N, Abdolell M, Warde P, Toi A, Catton C (2001). Positioning errors and prostate motion during conformal prostate radiotherapy using on-line isocentre set-up verification and implanted prostate markers. Radiother Oncol.

[B17] Ullman KL, Ning H, Susil RC, Ayele A, Jocelyn L, Havelos J, Guion P, Xie H, Li G, Arora BC, Cannon A, Miller RW, Coleman CN, Camphausen K, Menard C (2006). Intra- and inter-radiation therapist reproducibility of daily isocenter verification using prostatic fiducial markers. Radiation Oncology.

[B18] Berthelet E, Liu MCC, Agranovich A, Patterson K, Currie T (2002). Computed tomography determination of prostate volume and maximum dimensions: a study of interobserver variability. Radiother Oncol.

[B19] Cazzaniga LF, Marinoni MA, Bossi A, Bianchi E, Cagna E, Cosentino D, Scandolaro L, Valli M, Frigerio M (1998). Interphysician variability in defining the planning target volume in the irradiation of prostate and seminal vesicles. Radiother Oncol.

[B20] Bissett R, Boyko S, Leszczynski K, Cosby S, Dunscombe P, Lightfoot N (1995). Radiotherapy portal verification: an observer study. Br J Radiol.

[B21] Ludbrook JJ, Greer P, Blood P, D'yachkova Y, Coldman A, Beckham WA, Runkel J, Olivotto IA (2005). Correction of systematic setup errors in prostate radiation therapy: how many images to perform?. Med Dosim.

